# Low-Frequency *PPM1D* Gene Mutations Affect Treatment Response to BCMA-Targeted CAR T-Cell Therapy in Multiple Myeloma

**DOI:** 10.3390/cancers18132032

**Published:** 2026-06-23

**Authors:** Katharina van der Weg, Martina Bertschinger, Ulrike Bacher, Michele Hoffmann, Henning Nilius, Katja Seipel, Thomas Pabst

**Affiliations:** 1 Department of Medical Oncology, Inselspital, Bern University Hospital, University of Bern, 3010 Bern, Switzerland; katharina.vanderweg@students.unibe.ch (K.v.d.W.); martina.bertschinger@insel.ch (M.B.); michele.hoffmann@insel.ch (M.H.); 2 Department of Hematology, Inselspital, Bern University Hospital, 3010 Bern, Switzerland; veraulrike.bacher@insel.ch; 3 Department of Clinical Chemistry, Inselspital, Bern University Hospital, 3010 Bern, Switzerland; 4 Department for Biomedical Research (DBMR), University of Bern, 3008 Bern, Switzerland; katja.seipel@unibe.ch

**Keywords:** *PPM1D*, myeloma, CAR T-cell, clonal hematopoiesis, outcome, survival

## Abstract

**Background**: BCMA-targeted Chimeric Antigen Receptor (CAR) T-cell therapy has revolutionized the treatment of Relapsed/Refractory Multiple Myeloma (RRMM). However, the disease is not curable and progression after CAR T-cell treatment remains a challenge. Clonal hematopoiesis, specifically mutations in the DNA damage response gene *PPM1D*, has been linked to therapy resistance and inferior survival in lymphoma patients undergoing cellular therapy. The impact of *PPM1D* mutations on MM patient outcome after CAR T-cell therapy remains undefined. **Methods**: We conducted a retrospective single-center study of 83 patients with RRMM patients treated with idecabtagene vicleucel or ciltacabtagene autoleucel between 2022 and 2025. Next-generation sequencing was performed on peripheral blood mononuclear cells collected prior to CAR T-cell infusion to identify *PPM1D* exon 6 mutations (variant allele frequency > 0.01). We analyzed associations between mutational status, clinical characteristics, toxicity, and survival. **Results**: *PPM1D* mutations were detected in 14.5% (12/83) of patients. *PPM1D*-mutated patients had fewer prior autologous stem cell transplantation compared to wild-type patients (50% vs. 82%, *p* = 0.02) and presented more advanced disease burden and adverse prognostic features (R-ISS stage III 58% vs. 20%, *p* = 0.05). Notably, *PPM1D* status did not impact initial efficacy; complete remission rates were comparable between groups (67% vs. 69%). However, *PPM1D* mutations were significantly associated with inferior progression-free survival (PFS) (median PFS: 6 months vs. 16 months, *p* = 0.04). Regarding toxicity, the mutated subgroup exhibited significantly higher rates of grade ≥2 cytokine release syndrome and a trend toward increased neurotoxicity (25% vs. 7%). **Conclusions**: *PPM1D* clonal hematopoiesis is frequent in RRMM and despite deep initial responses, patients harboring *PPM1D* mutations face a significantly higher risk of early relapse. *PPM1D* mutations may serve as a biomarker for poor durability of response and should be further evaluated in larger, prospective trials.

## Simple Summary

Multiple Myeloma is an incurable plasma cell disorder, which, despite major therapeutic advances, particularly with the development of CAR T-cell therapy, remains incurable. Clonal hematopoiesis with mutations in the *PPM1D* gene can develop after repeated treatments. These mutations have previously been linked to treatment resistance and poor survival in lymphoma patients receiving cellular therapies. This retrospective study analyzed patients treated with CAR T-cell therapy between 2022 and 2025 to evaluate the impact of *PPM1D* mutations on treatment outcomes. Genetic screening before CAR T-cell infusion assessed mutation status, which was correlated with clinical characteristics, toxicity, and survival. *PPM1D* mutations were detected in 14.5% of the relapsed refractory Multiple Myeloma patients. Mutated patients showed more advanced disease burden and adverse prognostic features and fewer prior high-dose chemotherapy. While mutation status did not affect initial response depth, it was associated with shorter treatment durability after CAR T-cell therapy.

## 1. Introduction

Multiple Myeloma (MM) is a clonal plasma cell malignancy that accounts for about 10% of all hematological malignancies [1]. Over the past two decades, the introduction of novel agents including proteasome inhibitors (PIs), immunomodulatory drugs (IMiDs) and monoclonal antibodies (mAbs) has led to substantial improvements in outcomes for patients with newly diagnosed MM [2,3,4]. Standard first-line therapy typically consists of an induction regimen including a PI, an IMiD, and a mAb, followed by high-dose chemotherapy with autologous stem cell transplantation (HDCT/ASCT) in eligible patients [3,4]. Despite all the advantages, MM remains incurable, and almost all patients with MM eventually relapse [4]. The management of patients with relapsed or refractory MM (RRMM) remains a significant clinical challenge. With successive lines of therapy, the duration of response has shown to decrease with each therapy line [5]. In particular, patients refractory to multiple drug classes have limited treatment options and a poor prognosis [6,7,8].

Chimeric antigen receptor (CAR) T-cell therapy has emerged as a novel immunotherapeutic approach for the treatment of RRMM. The clinically most advanced CAR T-cell products in RRMM target B-cell maturation antigen (BCMA), a surface antigen predominantly expressed on differentiated B-cells including malignant plasma cells [9]. Currently, two BCMA-targeted CAR T-cell products, idecabtagene vicleucel (ide-cel, Abecma^®^) and ciltacabtagene autoleucel (cilta-cel, Carvykti^®^), have received regulatory approval by the European Medicines Agency (EMA) for patients with RRMM who have received at least one prior line of therapy. Both products have demonstrated substantial clinical activity in heavily pretreated RRMM with median progression-free survival (PFS) of approximately 12 months for ide-cel and exceeding 30 months for cilta-cel [10,11,12,13,14,15,16,17,18,19]. Notably, the observed benefit was consistent even through high-risk subgroups [13,18,20]. Despite its efficacy, CAR T-cell therapy is frequently associated with clinically relevant toxicities, including cytokine release syndrome (CRS), immune effector cell-associated neurotoxicity syndrome (ICANS), infections and prolonged cytopenias [21]. CRS and ICANS are managed according to established grading systems, with tocilizumab as first-line therapy for CRS and corticosteroids in cases of neurotoxicity or higher-grade CRS [22]. Across clinical trials, CRS has been reported in 75–95% of RRMM patients after CAR T-cell infusion and neurotoxicity in 15–42%, with the majority of events being grade 1 or 2 [11,12,13,15,17,18,23]. Hematologic toxicities, such as neutropenia, leukopenia, anemia and thrombocytopenia, represent the most frequent higher-grade (≥3) adverse events [13,15,18]. In indirect comparison of ide-cel and cilta-cel, Hansen et al. reported superior efficacy and survival with cilta-cel, albeit at the cost of higher rates of certain toxicities, including higher-grade CRS, infections, second primary malignancies, and delayed neurotoxicity [24]. Recently, studies including less heavily pretreated patients have demonstrated improved treatment responses and lower toxicity rates, supporting the evaluation of CAR T-cell therapy in earlier treatment lines [10,15,19,25].

Clonal hematopoiesis (CH) refers to the clonal expansion of hematopoietic stem cells harboring somatic mutations in the absence of overt hematologic malignancy, typically defined by a variant allele frequency (VAF) of ≥0.02. The prevalence of CH increases with age and is further enriched in patients with cancer, particularly following exposure to cytotoxic chemotherapy and radiotherapy [26,27]. CH is driven by mutations in genes that provide a selective advantage to hematopoietic stem cells. *PPM1D*, a gene involved in the DNA damage response (DDR), is a driver gene of CH [27,28]. The *PPM1D* gene is located on chromosome 17q and encodes the phosphatase Mg2+/Mn2+-dependent 1D protein (*PPM1D*, Wip1), a serin/threonine phosphatase that is transcriptionally upregulated in a p53-dependent manner in response to cellular stress, including DNA damage [29,30]. *PPM1D* negatively regulates p53 and other DDR proteins, thereby promoting termination of the DDR and restoration of cellular homeostasis [31]. By attenuating p53 signaling, *PPM1D* protects cells from apoptosis or senescence under genotoxic stress [32,33]. As long as *PPM1D* is not overexpressed, its role appears to be benign and vital in stem cell homeostasis [31,34]. In hematopoietic cells, *PPM1D* alterations predominantly consist of nonsense or frameshift mutations clustered in exon 6, resulting in C-terminally truncated protein products with preserved phosphatase activity and increased protein stability due to impaired proteasomal degradation [35,36]. These gain-of-function mutations lead to resistance to apoptosis under DNA-damaging conditions [37]. Multiple studies have demonstrated strong associations between *PPM1D* mutations and prior exposure to cytotoxic chemotherapy as well as radiotherapy, indicating therapy-driven clonal selection of *PPM1D*-mutated cells [26,30,38,39]. Consistently, *PPM1D*-mutant cells exhibit increased resistance to chemotherapy, likely mediated by enhanced protein stability and reduced apoptotic susceptibility [30,40].

CH is frequently detected in patients with lymphoma or MM undergoing CAR T-cell therapy or HDCT/ASCT, with reported prevalences ranging from 21% to over 50% [41,42,43]. In the HDCT/ASCT setting, CH—particularly involving DDR genes such as *PPM1D*—has been linked to inferior clinical outcomes, including faster disease progression, inferior survival and an increased risk of therapy-related myeloid neoplasms [42,44,45,46]. In MM patients undergoing HDCT/ASCT, truncating *PPM1D* mutations were more frequently detected after repeated transplantation and were associated with inferior PFS and OS [47]. We have previously demonstrated inferior survival outcomes following CD19-targeted CAR T-cell therapy in patients with large B-cell and mantle cell lymphoma harboring *PPM1D* mutations [48,49].

In this retrospective study, we determined the prevalence of *PPM1D* exon 6 mutations in peripheral blood mononuclear cells (PMBCs) of patients with MM undergoing BCMA-targeted CAR T-cell therapy and analyzed their association with clinical outcomes. Using next-generation sequencing (NGS) amplicon analysis, we evaluated correlations between *PPM1D* mutational status and treatment response, survival outcomes and therapy-related toxicities.

## 2. Materials and Methods

We conducted a retrospective single-center study at the Inselspital, University Hospital of Bern, Switzerland. The final study cohort comprised 83 patients with triple-class exposed RRMM, who received commercially available CAR T-cell therapy between June 2022 and October 2025. All patients provided written informed consent for the use of their personal data for research purposes. Clinical follow-up evaluations were scheduled at 3 and 6 months, at 1 year and annually thereafter following CAR T-cell infusion. In addition, clinical and laboratory data related to both the underlying disease and CAR T-cell therapy were collected.

Genomic DNA was extracted from mononuclear cells (PBMCs) isolated from the peripheral blood of 83 MM patients collected before CAR T-cell infusion and 8 healthy donors. NGS amplicon sequencing was performed. Library preparation included the construction of a Nextera two-step PCR library, followed by sequencing on an Illumina MiSeq platform using a MiSeq Reagent Kit v3 (600-cycle) with 2 × 300 bp paired-end reads (Ilumina, San Diego, CA, USA). Gene-specific primers covering exon 6 of the *PPM1D* gene were used (forward: 5′-GAGGATCCATGGCCAAGGG-3′; reverse: 5′-TTCCAATTTTCTTCTGGCCCC-3′; amplicon size: 505 bp). Sequencing was performed by Microsynth (Balgach, Switzerland).

Bioinformatic analysis included trimming of locus-specific Illumina adapter sequences, merging of paired-end reads, mapping of the trimmed and merged reads to human chromosome 17 for variant calling and annotation, and mapping to the selected PPM1D region (chr17:60,662,994–60,663,549) for coverage analysis. In addition, trimmed and merged reads were dereplicated. A total of 2,586,264 demultiplexed reads passed Illumina’s chastity filter, corresponding to 777,838,210 demultiplexed bases, with a mean read length of 301 bp. FastQC (version 0.11.9) was used to assess quality. The quality assessment returned a mean Q of 35, with 95% in Q20 and 89% in Q30. The method was described in detail by Seipel et al. [47]. As described in Seipel et al. the VAF of *PPM1D* gene mutations called in the DNA samples ranged from 0.01 to 0.05 [47]. We therefore applied a cutoff of 0.01 in the current study. NGS amplicon sequencing and bioinformatics followed previous descriptions [47,48].

The primary endpoints were survival outcomes. PFS and overall survival (OS) were defined as the time from CAR T-cell infusion to disease progression, death, or last follow-up, respectively. PFS and OS were censored at the last follow-up on 15 December 2025, which was also used as the data cutoff.

Parameters investigated for their potential prognostic significance were patient age, R-ISS stage, cytogenetic risk, number of prior treatment lines including HDCT/ASCT, prior radiotherapy, the need for bridging therapy before CAR T-cell infusion, remission status before and after CAR T-cell treatment, use of Abecma^®^ or Carvykti^®^ CAR T-cell products, the manifestation of a CRS and ICANS.

Kaplan–Meyer survival curves and univariate statistical analyses were performed using GraphPad Prism version 10 (GraphPad Software, San Diego, CA, USA). Categorical variables were summarized as frequencies and percentages, while continuous variables were reported as medians and ranges. Associations between categorical variables were analyzed using Fisher’s exact test. Comparisons of continuous variables between groups were performed using the Mann–Whitney U test. Survival differences between groups were assessed using the log-rank test. All statistical tests were two-sided. *p* values < 0.05 were considered significant.

Univariable and multivariable Cox proportional-hazards models were additionally fitted using the survival package (version 4.5.1) in R [50]. The multivariable models were adjusted for age, sex, R-ISS stage, high-risk cytogenetics, CAR-T product, and prior ASCT.

## 3. Results

### 3.1. Prevalence of PPM1D Mutations

NGS amplicon sequencing was performed to identify mutations in exon 6 of the *PPM1D* gene in PBMC obtained from 83 patients with MM prior to infusion of BCMA-targeted CAR T-cell therapies. Only *PPM1D* gene mutations with a VAF of >0.01 were included. Overall, 12 low-frequency *PPM1D* mutations were identified in 12 of 83 patients (14.5%), comprising 4 in-del, 7 nonsense and 1 missense mutations (Table 1). VAF of *PPM1D* mutations detected in PBMC-derived DNA samples ranged from 0.011 to 0.069 with a mean VAF of 0.032 and a median VAF of 0.017.

### 3.2. Baseline Clinical Characteristics

All 83 MM patients with available NGS data were admitted to CAR T-cell infusion. Clinical characteristics of the 83 patients are summarized in Table 2.

The median age at initial diagnosis was 58 years (range: 33–78) and the median age at CAR T-cell infusion was 67 years (range: 42–84), with no statistically significant difference between the *PPM1D*wt and *PPM1D*mut group. At initial diagnosis, Revised International Staging System (R-ISS) distribution differed between the subgroups, with a higher proportion of R-ISS stage III stage disease among *PPM1D*mut patients compared with *PPM1D*wt patients (58% vs. 20%, *p* = 0.05). Cytogenetic risk categories were similarly distributed between the groups, with high-risk cytogenetics observed in 42% of *PPM1D*mut and 32% of *PPM1D*wt patients (*p* = 0.68). Most patients were heavily pretreated prior to CAR T-cell therapy, with 60% of the overall cohort having received four or more prior lines of therapy, without differences between the *PPM1D* subgroups. Prior radiotherapy and the use of bridging therapy were comparable between *PPM1D*wt and *PPM1D*mut patients. In contrast, a significantly lower proportion of *PPM1D*mut patients had undergone HDCT/ASCT compared with *PPM1D*wt patients (50% vs. 82%, respectively; *p* = 0.02).

### 3.3. Disease Features and CAR T-Cell Treatment

Disease status at the time of CAR T-cell infusion and treatment-related characteristics are summarized in Table 3.

The median interval from initial diagnosis to CAR T-cell infusion was 76 months (range: 8–349 months) in the overall cohort, with no significant difference between the *PPM1D*wt and *PPM1D*mut group (82 vs. 66 months, *p* = 0.14). Disease status at infusion was comparable between subgroups, with progressive disease (PD) being the most frequent disease state in both *PPM1D*wt (45%) and *PPM1D*mut (50%) patients. As lymphodepleting chemotherapy, patients received either fludarabine/cyclophosphamide or bendamustine. Two different CAR T-cell products were used: Abecma^®^ (Brisol Myers Squibb, Princeton, NJ, USA, idecabtagen vicleucel; 57%) and Carvykti^®^ (Johnson & Johnson, New Brunswick, NJ, USA, ciltacabtagene autoleucel; 43%). Both the lymphodepleting chemotherapy regimens and the CAR T-cell products were used in comparable proportions across both subgroups.

### 3.4. Clinical Outcomes After CAR T-Cell Therapy

**Figure 1:**
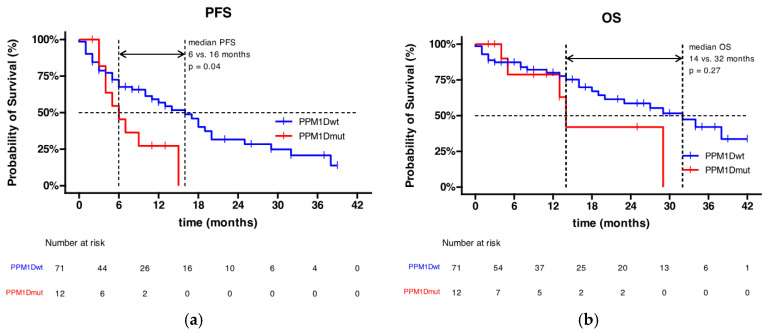
Clinical outcomes in RRMM patients treated with BCMA-targeted CAR T-cell therapy. (**a**) PFS and (**b**) OS of RRMM patients receiving BCMA-targeted CAR T-cell therapy were analyzed using Kaplan–Meyer, stratified by *PPM1D* status.

Clinical outcomes after CAR T-cell therapy are summarized in Table 4. CRS occurred in 87% of patients and was predominantly low grade. While CRS was more frequently observed in *PPM1D*wt patients (90% vs. 67%), the distribution of CRS grades differed significantly between the subgroups (*p* = 0.04), with a higher proportion of grade 1 CRS in *PPM1D*wt patients and a higher frequency of grade ≥2 CRS in the *PPM1D*mut subgroup. ICANS was observed in 10% of the overall cohort and occurred more frequently in *PPM1D*mut patients (25% vs. 7%, *p* = 0.07). Rates of admission to intermediate or intensive care units were low and comparable between subgroups. The median duration of hospitalization was similar in *PPM1D*wt and *PPM1D*mut patients (17 vs. 15.5. days, *p* = 0.24). Best response after CAR T-cell therapy did not differ between groups, with complete remission (CR) achieved in 69% of *PPM1D* and 67% of *PPM1D*mut patients. Relapse or disease progression occurred in 39% of *PPM1D*wt and 50% of *PPM1D*mut patients (*p* = 0.54). Survival outcomes favored the *PPM1D*wt subgroup, with significantly longer PFS (16 vs. 6 months, *p* = 0.04) and numerically longer OS (14 vs. 32 months, *p* = 0.27) compared to the *PPM1D*mut patients (Figure 1).

In the multivariable Cox proportional-hazards model, *PPM1D* mutation was an independent risk factor for PFS (HR = 2.58, 95% CI 1.02–6.52, *p* = 0.044) but not for OS (HR = 1.78, 95% CI 0.50–6.34, *p* = 0.4) (Table S1).

## 4. Discussion

In this retrospective single-center study, we investigated the prevalence and clinical impact of *PPM1D* exon 6 mutations in patients with RRMM undergoing BCMA-targeted CAR T-cell therapy. To our knowledge, this is one of the first studies to specifically assess the prognostic value of *PPM1D* CH in the context of CAR T-cell therapy for MM. Our primary finding is that *PPM1D* mutations are present in a relevant proportion of this heavily pretreated patient population and are associated with significantly shorter PFS and a trend toward increased immunotoxicity, despite comparable initial treatment response rates.

The observed prevalence of *PPM1D* mutations in our cohort was 14.5%, which is slightly lower than rates reported in some broader CH studies involving lymphoma and MM patients [41,42,43]. This frequency underscores that *PPM1D*-mutant clones are a recurring feature of the hematopoietic landscape in patients exposed to chronic cytotoxic stress. Importantly, the presence of these mutations correlated with significantly shorter median PFS (6 months vs. 16 months for wild-type patients).

The impact of *PPM1D* mutations on clinical outcomes after CAR T-cell therapy has been documented across different hematological diseases. Notably, an evaluation of 85 patients with relapsed/refractory diffuse large B-cell lymphoma undergoing CD19-targeted CAR T-cell therapy revealed a *PPM1D* mutation prevalence of 20%; within this cohort, patients harboring these mutations exhibited significantly shorter PFS and OS [48]. This negative prognostic association was further elaborated by Seipel et al. in patients with mantle cell lymphoma receiving CD19-directed CAR T-cell treatment. This retrospective analysis similarly identified *PPM1D* mutations as a determinant of inferior survival outcomes [49]. These data concur with our present findings in patients with RRMM. The reproducibility of this negative prognostic signal across different hematologic malignancies and CAR T-cell targets suggests that *PPM1D* may represent a pan-disease biomarker of poor long-term outcomes in patients treated with CAR T-cell therapy.

We observed a dissociation between initial response and durability. We found no significant difference in the best overall response or CR rates between *PPM1D*-mutated and wild-type patients (67% vs. 69% CR rates). This suggests that the presence of *PPM1D*-mutant clones in the peripheral blood does not impair the immediate cytotoxic efficacy of the CAR T-cell product. However, the rapid relapse rate in the mutated cohort implies that *PPM1D* mutations may be indicative of an unfavorable host environment that fails to sustain remission or perhaps correlates with a more aggressive underlying myeloma biology, as suggested by the higher proportion of R-ISS stage III disease in the mutated group.

Mechanistically, the impact of *PPM1D* mutations on CAR T-cell therapy is likely multifactorial. Since CAR T-cells are autologous products manufactured from PBMCs, it is plausible that a fraction of the infused T-cells carries the *PPM1D* mutation.

While *PPM1D* truncating mutations confer apoptosis resistance—which theoretically could enhance T-cell persistence—they also dampen p53-mediated DNA damage responses [31,32,33,34]. This could lead to genomic instability within the T-cell compartment or functional exhaustion. Alternatively, *PPM1D* mutations in the myeloid compartment (monocytes/macrophages) may contribute to a pro-inflammatory microenvironment, which leads to immunosuppression. CH is well-documented to drive aberrant inflammation via the NLRP3 inflammasome [51,52]. Our toxicity data supports this “inflammatory host” hypothesis: *PPM1D*-mutated patients exhibited a significant shift toward higher-grade CRS and a trend toward higher rates of ICANS (25% vs. 7%). This suggests that *PPM1D* clones may amplify the cytokine storm associated with CAR T-cell engagement, potentially worsening the therapeutic index.

Interestingly, our study revealed a counter-intuitive inverse relationship between prior HDCT/ASCT and *PPM1D* status. While findings from other studies suggest that HDCT/ASCT exert selective pressure favoring *PPM1D* mutant expansion, our mutated cohort had significantly fewer prior HDCT/ASCTs compared to the wild-type group (50% vs. 82%) [42,44,47]. This discrepancy might be explained by clinical selection bias; patients harboring *PPM1D* mutations presented with higher-risk disease (R-ISS III) and may have been deemed ineligible for transplant earlier in their disease course due to comorbidities or refractory disease, leading to reliance on other alkylating agents or continuous therapy lines that drove clonal selection. Alternatively, this may indicate that in the RRMM setting, the cumulative dose of novel agents and continuous chemotherapy may select for *PPM1D* clones as effectively as single-event HDCT/ASCT.

This study has several limitations. First, the retrospective, single-center design and the relatively small number of patients with *PPM1D* mutations (*n* = 12) limit the statistical power for subgroup analyses, particularly regarding OS, which showed a numerical but non-significant trend. Due to the limited sample size, the results should be seen as exploratory and confirmed in a larger cohort. Second, we performed targeted sequencing of PBMCs without sorting specific cell lineages; therefore, we cannot definitively state whether the mutations reside primarily in the myeloid compartment, the T-cell compartment (including the CAR T-cells themselves), or residual circulating plasma cells, although the latter is less likely given the VAF thresholds used. Another limitation of this analysis is that only *PPM1D* mutations were evaluated. Other CHIP-associated mutations were not captured and could likewise have contributed to the observed associations, potentially influencing the results. Additionally, as we used unsorted PBMCs, the cellular origin of the mutations remains unknown. Since CAR T-cells are autologous products manufactured from PBMCs, it is plausible that a fraction of the infused T-cells carries the *PPM1D* mutation. Finally, the heterogeneity of prior treatment lines and the use of two different CAR T-cell products (ide-cel and cilta-cel) introduce variability, although distribution was balanced between groups.

## 5. Conclusions

*PPM1D* mutations are frequent in RRMM patients treated with CAR T-cell therapy, and in our analysis, they are associated with inferior PFS and increased toxicity. Unlike markers of primary refractory disease, *PPM1D* status does not appear to hinder initial remission induction, but in our observational study, it is associated with a lack of durable disease control. These findings should be evaluated in future larger studies.

## Figures and Tables

**Table 1:** *PPM1D* gene mutations detected in MM cohort.

**Classification**	**Locus**	**VAF**	**NT Change**	**AA Change**
nonsense	chr17:60,6663352	0.011	G/T	E540 *
nonsense	chr17:60,6663292	0.013	C/T	Q520 *
nonsense	chr17:60,6663182	0.018	T/G	L484 *
nonsense	chr17:60,6663077	0.013	T/G	L450 *
nonsense	chr17:60,6663136	0.012	C/A	S468 *
nonsense	chr17:60,6663345	0.015	T/G	L538 *
nonsense	chr17:60,6663158	0.014	G/T	E475 *
indel	chr17:60,6663077	0.069	AT/A	L450fs
indel	chr17:60,6663310	0.039	GA/G	I526fs
missense	chr17:60,6663086	0.071	G/A	E451K
indel	chr17:60,6663040	0.052	TA/T	R536fs
indel	chr17:60,6663273	0.062	AT/A	L513fs

* nonsense mutation.

**Table 2:** Baseline clincal characteristics of the RRMM cohort, univariate analysis.

	Cohort (*n* = 83)	*PPM1D*wt (*n* = 71)	*PPM1D*mut (*n* = 12)	*p*-Value
Sex (female:male)	26:57	23:48	3:9	0.74
Median age at ID (years)	58 (33–78)	58 (33–78)	63.5 (43–76)	0.08
Median age at CAR T (years)	67 (42–84)	66 (42–82)	70.5 (50–84)	0.16
R-ISS stage at ID				0.05
I	21 (25%)	19 (27%)	2 (17%)	
II	33 (40%)	31 (44%)	2 (17%)	
III	21 (25%)	14 (20%)	7 (58%)	
no info	8 (10%)	7 (10%)	1 (8%)	
Cytogenetic abnormalities				0.68
standard risk	32 (39%)	27 (38%)	5 (42%)	
high risk	28 (34%)	23 (32%)	5 (42%)	
no info	23 (28%)	21 (30%)	2 (17%)	
Extramedullary disease	17 (20%)	15 (21%)	2 (17%)	>0.99
Number of treatment lines prior to CAR T-cell therapy				>0.99
1–3	33 (40%)	28 (39%)	5 (42%)	
≥4	50 (60%)	43 (61%)	7 (58%)	
Radiotherapy	44 (53%)	36 (51%)	8 (67%)	0.36
Prior HDCT/ASCT	64 (77%)	58 (82%)	6 (50%)	0.02
Prior BCMA exposure	7 (8%)	6 (8%)	1 (8%)	>0.99
Bridging Therapy	48 (58%)	41 (58%)	7 (58%)	>0.99

ID = initial diagnosis, R-ISS = Revised International Staging System, HDCT/ASCT = High-dose chemotherapy with autologous stem cell transplant, BCMA = B-cell maturation antigen.

**Table 3:** Clinical characteristics and details of CAR T-cell treatment, univariate analysis.

	Cohort (*n* = 83)	*PPM1D*wt (*n* = 71)	*PPM1D*mut (*n* = 12)	*p*-Value
LD Chemotherapy				0.35
Flu/Cy	40 (48%)	36 (51%)	4 (33%)	
Bendamustine	43 (52%)	35 (49%)	8 (67%)	
CAR T-cell product				0.76
Abecma^®^	47 (57%)	41 (58%)	6 (50%)	
Carvykti^®^	36 (43%)	30 (42%)	6 (50%)	
Stage at CAR T-cell infusion				>0.99
CR	6 (7%)	5 (7%)	1 (8%)	
PR	21 (25%)	18 (25%)	3 (25%)	
SD	18 (22%)	16 (23%)	2 (17%)	
PD	38 (46%)	32 (45%)	6 (50%)	
Median interval ID to CAR T-cell infusion (months)	76 (8–349)	82 (11–224)	66 (8–349)	0.14

LD = lymphodepleting, Flu/Cy = fludarabine/cyclophosphamide, CR = complete response, PR = partial response, SD = stable disease, PD = progressive disease, ID = initial diagnosis.

**Table 4:** Clinical outcome after CAR T-cell treatment, univariate analysis.

	Cohort (*n* = 83)	*PPM1D*wt (*n* = 71)	*PPM1D*mut (*n* = 12)	*p*-Value
CRS	72 (87%)	64 (90%)	8 (67%)	0.04
grade 1	58 (70%)	53 (75%)	5 (42%)	
grade 2	13 (16%)	10 (14%)	3 (25%)	
grade 3	0 (0%)	0 (0%)	0 (0%)	
grade 4	1 (1%)	1 (1%)	0 (0%)	
ICANS	8 (10%)	5 (7%)	3 (25%)	0.07
grade 1	3 (4%)	1 (1%)	2 (17%)	
grade 2	3 (4%)	2 (3%)	1 (8%)	
grade 3	0 (0%)	0 (0%)	0 (0%)	
grade 4	2 (2%)	2 (3%)	0 (0%)	
Admissions to IMC/ICU	7 (8%)	6 (8%)	1 (8%)	>0.99
Median Hospitalization time (days)	17 (8–93)	17 (8–93)	15.5 (9–21)	0.24
Best remission status post CAR T-cell therapy				0.64
CR	57 (69%)	49 (69%)	8 (67%)	
PR	11 (13%)	9 (13%)	2 (17%)	
SD	3 (4%)	2 (3%)	1 (8%)	
PD	12 (14%)	11 (15%)	1 (8%)	
Relapse/Progression	34 (41%)	28 (39%)	6 (50%)	0.54
Median survival time				
PFS (months)	14	16	6	0.04
OS (months)	29	32	14	0.27

CRS = cytokine release syndrome, ICANS = immune effector cell-associated neurotoxicity syndrome, IMC = intermediate care unit, ICU = intensive care unit, CR = complete response, PR = partial response, SD = stable disease, PD = progressive disease, PFS = progression free survival, OS = overall survival.

## Data Availability

Deidentified individual participant data are available from the corresponding author upon reasonable request.

## Supplementary Materials

The following supporting information can be downloaded at: https://www.mdpi.com/article/10.3390/cancers18132032/s1, Table S1: Univariate and multivariate analysis. ID = initial diagnosis, R-ISS = Revised International Staging System, ASCT = High-dose chemotherapy with autologous stem cell transplant.

## Abbreviations

The following abbreviations are used in this manuscript:

BCMA	B-Cell maturation antigen
CAR	Chimeric antigen receptor
RRMM	Relapsed/refractory multiple myeloma
*PPM1D*	Protein phosphatase Mg2+/Mn2+-dependent 1D
MM	Multiple myeloma
R-ISS	Revised International Staging System
PFS	Progression-free survival
OS	Overall survival
PI	Proteasome inhibitor
IMiD	Immunomodulatory drugs
mAb	Monoclonal antibodies
HDCT	High-dose chemotherapy
ASCT	Autologous stem cell transplantation
CRS	Cytokine release syndrome
ICANS	Immune effector cell-associated neurotoxicity syndrome
CH	Clonal hematopoiesis
VAF	Variant allele frequency
DDR	DNA damage response
PMBC	Peripheral blood mononuclear cell
NGS	Next generation sequencing
wt	Wild type
mut	Mutated
