# Supplementary material for: Low-Frequency PPM1D Gene Mutations Affect Treatment Response to BCMA-Targeted CAR T-Cell Therapy in Multiple Myeloma
Source: Cancers (Basel). 2026 Jun 23;18(13):2032. doi: 10.3390/cancers18132032 (PMC13359572; doi:10.3390/cancers18132032)
Supplement: Supplementary file 1 [file cancers-18-02032-s001.zip › cancers-4357841-supplementary.pdf]

Supplementary Table S1: Univariate and multivariate analysis.

Characteristics	Univariate regression				Multivariate regression			
	PFS		OS		PFS		OS	
	HR	p-value	HR	p-value	HR	p-value	HR	p-value
***PPM1D* status**								
Wild type	–	–	–	–	–	–	–	–
Mutated	2.15	0.046	1.74	0.3	2.85	0.044	1.78	0.4
**Age**	1.00	0.8	1.01	0.5	0.98	0.5	1.00	>0.9
**Sex**								
Female	–	–	–	–	–	–	–	–
Male	0.68	0.2	0.77	0.5	0.56	0.1	0.67	0.4
**R-ISS stage ad ID**								
I	–	–	–	–	–	–	–	–
II	1.11	0.8	2.00	0.2	1.07	0.9	2.03	0.2
III	1.82	0.13	1.70	0.3	1.48	0.4	1.42	0.6
**Cytogenetic risk**								
No information	–	–	–	–	–	–	–	–
Standard risk	1.27	0.5	0.96	>0.9	1.40	0.4	0.98	>0.9
High risk	1.84	0.11	1.26	0.6	2.23	0.1	1.26	0.7
**CAR-T treatment**								
Ide-cel	–	–	–	–	–	–	–	–
Cilta-cel	0.58	0.14	0.52	0.3	0.59	0.2	0.60	0.4
**Prior ASCT**								
ASCT	–	–	–	–	–	–	–	–
No ASCT	1.12	0.8	1.28	0.6	0.52	0.2	0.97	>0.9

ID = initial diagnosis, R-ISS = Revised International Staging System, ASCT = High-dose chemotherapy with autologous stem cell transplant
